# Assessment of the gifted adolescents’ functional state of the organism under the psychological stress

**DOI:** 10.1192/j.eurpsy.2022.765

**Published:** 2022-09-01

**Authors:** M. Arakelyan

**Affiliations:** Yerevan State Medical University after Mkhitar Heratsi, Medical Psychology, Yerevan, Armenia

**Keywords:** Gifted adolescents, functional state, Stress Index

## Abstract

**Introduction:**

Many studies have shown that gifted children and youth have difficulties in education, emotional regulations, psychological adjustment process etc.

**Objectives:**

Our aim is to evaluate the adaptive capacity, the functional state of the gifted adolescents’ organism under external potential stressor.

**Methods:**

The research has been conducted in schools of Yerevan, RA. The initial sample consisted of 500 high school students aged 16-18. Renzulli’s Three-Ring Conception of Giftedness was used to reveal gifted adolescents. In the course of study 35 of 500 participants were defined as gifted. The quasi-experimental design has been used with 35 participants in the comparison and experiment group each. For comparative analyses, we used Heart rate variability (HRV) method. As a potential stressor, the intellectual workload was selected. The ECG indicators have been recorded for 5 minutes each before and after the intellectual workload. We are presenting the results through Stress Index.

**Results:**

As we can see from the picture 1. the Stress Index (SI) of gifted girls and boys are higher from norm (the norm is 20-100). For control groups, the SI is within the norm. The SI for gifted groups of adolescents significantly higher from those of control groups. The data indicates, that for gifted adolescents the activity of central mechanisms prevails over autonomous mechanisms.

**Figure:**
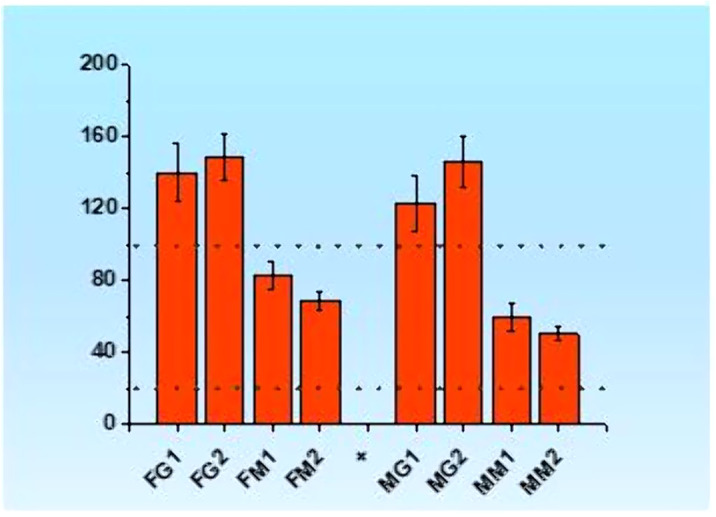

**Conclusions:**

The level of stress in gifted adolescents is higher than that of the control group and rises in case when the task wasn’t solved. High results speak about psycho-emotional tension and stress. Further research is needed to understand the psychological background of such reactions.

**Disclosure:**

No significant relationships.

